# The immune regulation of BCL3 in glioblastoma with mutated IDH1

**DOI:** 10.18632/aging.204048

**Published:** 2022-04-29

**Authors:** Shibing Fan, Na Wu, Shichuan Chang, Long Chen, Xiaochuan Sun

**Affiliations:** 1 Department of Neurosurgery, Chongqing Medical University, Chongqing, China; 2 Chongqing University Three Gorges Hospital, Wanzhou, Chongqing, China; 3 Chongqing University, Shapingba, Chongqing, China

**Keywords:** IDH1, glioblastoma, BCL3, immune microenvironment

## Abstract

Background: Glioblastoma in the brain is the most malignant solid tumor with a poor prognosis. Screening critical targets and exploring underlying mechanisms will be a benefit for diagnoses and treatment. IDH1 mutation (R132) was used to distinguish glioblastoma grade and predict prognosis as a significant marker. However, the manner of IDH1 mutation regulating glioblastoma development was still unclear.

Methods: To study the function of IDH1 mutation, multi-type sequencing data (transcriptome, methylation and copy number variation) from the GEO and TCGA database were analyzed using bioinformatics techniques. The biological functions of IDH1 mutation (R132) would be comprehensively evaluated from the regulatory networks, tumor immune microenvironment and clinical relevance. Then the analysis result would be validated by experimental techniques.

Results: Compared with adjacent tissues, IDH1 was up-regulated in glioblastoma, which also positively correlated with the malignant degree and a poor prognosis. To further study the mechanism of mutated IDH1 (R132) function, 5 correlated genes (FABP5, C1RL, MIR155HG, CSTA and BCL3) were identified by different expression gene screening, enrichment analysis and network construction successively. Among them, the BCL3 was a transcription factor that may induce IDH1expression. Through calculating the correlation coefficient, it was found that in IDH1^mut^ glioblastoma, the dendritic cell infiltration was reduced which may result in a better prognosis. In addition, the level of IDH1, FABP5, C1RL, MIR155HG, CSTA and BCL3 might also influence lymphocytes infiltration (eg. CD4+ T cell) and chemokine expression (CXCL family).

Conclusions: IDH1 may participate in pathological mechanisms of glioblastoma via expression alteration or gene mutation. Furthermore, IDH1 mutation might improve prognosis via suppressing the expression of FABP5, C1RL, MIR155HG, CSTA and BCL3. Meanwhile, it was identified that BCL3 might perform similar immunomodulatory functions with IDH1 as an upstream transcript factor.

## INTRODUCTION

Glioblastoma (GBM) is the most malignant and frequent cancer type in the central nervous system (CNS). Most GBM (~90%) develop rapidly in elderly patients. The unclear pathogenesis leads to therapeutic difficulty and high mortality. Despite the similar histologic appearance, the primary and secondary GBM are distinct cancers that originate from various precursor cells and require different therapeutic approaches. In 2008, the high-throughput sequencing of WHO grade I-IV gliomas were performed. In 12% of samples, a novel mutation at codon 132 (R132) of isocitrate dehydrogenase 1 (IDH1) was identified in ~80% of secondary GBM as a decisive genetic signpost [1–4]. Meanwhile, in primary GBM, this mutation was occurred rarely (<5%) [5, 6]. Previous studies have identified that in secondary GBM, the IDH1 (R132) mutation is a more objective and reliable diagnostic marker than clinical observation [7, 8]. The IDH1 (R132) mutation had never been linked to GBM before but now the mechanism of GBM development is under intense investigation.

IDH1 is the enzyme that performs key roles in various cellular functions, including the regulation of carbohydrate metabolism, epigenetics, differentiation, DNA repair, and redox states [9]. Dimers in the cytosol are essential for IDH1 enzymatic activity [10]. Dimeric IDH1 contains 2 active sites for catalyzing isocitrate to α-ketoglutarate (α-KG) by oxidative decarboxylation while generating reduced nicotinamide adenine dinucleotide phosphate (NADPH) from NADP+ [11]. The IDH1 R132 residue is almost heterozygous [9] and located in the isocitrate binding sites [4], which would suppress conversion from isocitrate to α-KG [12]. However, IDH1 R132 mutation in GBM is also a gain-of-function alteration resulting in hampered production and accelerated consumption of NADPH, which produces the 2-hydroxyglutarate (2-HG) from α-KG [13–15]. The concentration of 2-HG in malignant IDH1^mut^ GBMs would be increased about 100 folds [13]. Thus, mutant IDH1 could suppress the bioavailability of α-KG and competitively inhibit α-KG-dependent dioxygenases via increasing 2-HG, including histone demethylases and the TET family of 5-methylcytosine (5mC) hydroxylases, which mediate DNA demethylation [16]. As a result, gliomas with mutant IDH1 manifest a CpG island methylator phenotype (CIMP), which epigenetically alters gene expression by DNA hypermethylation [17]. 2-HG could also block prolyl-hydroxylation of collagen, leading to defect in collagen protein maturation affecting glioma progression [18]. Brain-specific IDH1 (R132H) conditional knock in mice exhibited hemorrhage and perinatal lethality [19]. In addition, it suggests that IDH1^mut^ glioma cells might have stem-cell-like features with growth advantage under neurosphere culture conditions [20].

B-cell CLL/lymphoma 3 (BCL-3), which belongs to the IκB family, can bind NF-κB homodimeric complexes of p50 or p52, which switches the transcriptional properties. BCL-3 overexpressed in breast cancer [21], nasopharyngeal carcinoma, endometrial cancer [22], hepatocellular carcinoma [23] and colorectal cancer [24] have been identified. Functionally, BCL-3 could participate in regulating the colony formation and cell cycle progression by regulating ubiquitination-mediated degradation of c-Myc in colorectal cancer [25]. Tu et al. [23] have reported that BCL3 promotes hepatocellular carcinoma growth by regulating cell proliferation and cell cycle through cyclin D1. However, the status and interaction with IDH1 of BCL3 in GBM have not been investigated.

In the present study, via bioinformatic analysis of GBM transcriptome sequencing datasets from GEO and TCGA databases and *in vivo* verification, try to find out the potential mechanism of IDH1 mutation of GBM development. Firstly, all samples were divided into wild type and IDH1-mutant groups. The different expression genes were identified according to |log FC|≥1.0 and p-value≤0.05 followed by enrichment analysis. In our study, via constructing a molecular network, the underlying mechanism of IDH1^mut^-BCL3 in GBM development was explored. The results laid a foundation for further studying the function of IDH1 mutation, and also provided theoretical support for better screening markers/targets and designing the therapeutic schedule.

## MATERIALS AND METHODS

### Sequencing datasets

On the one hand, the public GBM sequencing dataset (GSE122586) was obtained from the GEO database. According to the corresponding study [26], the GBM samples were collected in Beijing Tiantan Hospital from January 2005 to December 2009. The detailed sample information was introduced in Table 1. All surgically resected samples were rinsed with normal saline and then stored in liquid nitrogen immediately until use. All patients have voluntarily signed informed consent forms. The use of all human samples and the experimental procedures in that study were reviewed and approved by the Ethics Committee of Chinese Academy of Medical Sciences. In addition, another dataset GSE80729 contained U87 cells transfected with siRNA targeting BCL3 or control siRNA was used. The U87 glioma cell line is derived from a female patient with pleomorphic glioma. It differs from other glioma cell lines like U251 in cell proliferation, migration, and invasion. For example, U87 cells have exhibited a greater capacity for migration and invasion.

**Table 1:** The clinical data of GBM patients.

**IDH1 gene type**	**Wild type (n=73)**	**R132 mutation (n=16)**
**Age**	12~70 (Average=46.7)	27~62 (Average=40.7)
**Gender**	Male (n=43), Female (n=30)	Male (n=9), Female (n=7)
**Grade**	IV (n=73)	IV (n=16)
**DFS time(days)**	27~3293 (Average=491.8)	44~3478 (Average=1026.8)
**OS time(days)**	27~3293 (Average=644.4)	101~3478 (Average=1190.8)

On the other hand, the RNA-seq data (RSEM-normalized), methylation array data (Illumina Human Methylation 450) and CNV data (Affymetrix SNP 6.0) of GBMs from the TCGA database were downloaded from the NIH National Cancer Institute GDC Data Portal (https://portal.gdc.cancer.gov/). Overall survival (OS) was identified from the diagnosis date until death or the end of follow-up. In addition, disease-free survival (DFS) was defined as the period from diagnosis until the first disease progression with the clinical sign.

### Microarray data and enrichment analysis

Total RNA from cells and tissues was isolated using Trizol extractions (Invitrogen). The RNA quantity was assessed by NanoDrop®ND-1000 spectrophotometer (Agilent, Palo Alto, USA). 100 ng of total RNA was amplified using the Ambion® WT Expression Kit (4411973, Life Technologies). Then 5.5 μg of the cDNA was fragmented and labeled with the GeneChip® WT Terminal Labeling kit (901525, Affymetrix). Libraries were sequenced either on Illumina HiSeq 2000 or HiSeq 2500 using v3 chemistry.

Followed by background deletion, quantile normalization, and probe assembly. Different expression genes (DEGs) between normal vs. tumor tissues were detected by the empirical Bayes method [27]. The p-values were adjusted for multiple comparisons using the Benjamini-Hochberg procedure [28]. Genes with adjusted p-value < 0.05 and |log FC| ≥ 1.0 were considered as differentially expressed. Enrichment analysis of DEGs was performed with DAVID [29] and ClueGO [30]. The enriched GO (BP: biological process; CC: cellular component; MF: molecular function) and pathway terms were listed with participant genes [31]. Some other databases used are listed in Table 2.

**Table 2:** List of databases.

**Database ID**	**URL**
GEO Dataset	https://www.ncbi.nlm.nih.gov/gds/?term=
TCGA	https://www.cancer.gov/
cBioportal of cancer genomics	https://www.cbioportal.org/
TISIDB	http://cis.hku.hk/TISIDB/index.php
DSA	http://cancer.digitalslidearchive.net/
The Human Protein Atlas	https://www.proteinatlas.org/
STRING	https://string-db.org/
GEPIA	http://gepia.cancer-pku.cn/index.html
hTFtarget	http://bioinfo.life.hust.edu.cn/hTFtarget#!/

### Survival curves

Overall survival analyses were performed using the R package survival [32], and the patients were dichotomized based on the median expression. Kaplan-Meier estimator of survival was used to construct the survival curves. Log-rank tests (corresponding to a two-sided z test) were used to compare overall survival between patients in different groups. The hazard ratio (HR) (95% confidence interval) was provided for comparison of the two groups. The p-values were adjusted for multiple testing based on the false discovery rate (FDR) according to the Benjamini-Hochberg method [33]. Proportional hazard assumptions were tested.

### RT-qPCR

Total RNA was extracted using TRIzol® reagent (Takara Bio, Inc.). The concentration and purity of RNA were detected by NanoDrop®ND-1000 spectrophotometer. Total RNA was reverse transcribed into cDNA (10 μg) using the PrimeScript™ RT reagent kit (Takara Bio, Inc.), according to the manufacturer's protocol. The reaction conditions of the reverse transcription were: incubation at 25° C for 10 min, additional incubation at 42° C for 30 min, and heating at 95° C for 5 min. Then cDNA was diluted with DEPC water. Fluorescent RT-qPCR was performed according to the manufacturer's protocol. (Thermo Fisher Scientific, Inc.). The primers were designed and synthesized by Chongqing Life Biological Technology, Ltd. The reaction system volume was 25 μL consisting with cDNA (1 μL), 10X PCR buffer (2.5 μL), 10 mmol/l dNTPs (2 μL), PCR upstream primers (1 μL), PCR downstream primers (1 μL), Taq DNA polymerase (1 μL), and deionized water (16.5 μL). The reaction conditions were as follows: pre-denaturation at 95° C for 5 min, denaturation at 94° C for 1 min, annealing at 54° C for 45 sec, extension at 72° C for 1 min, in a total of 30 cycles, followed by extension at 72° C for 10 min. β-actin was used as the internal reference. The experiment was repeated 3 times independently.

### Immunohistochemistry (IHC)

Before IHC staining, glioma specimens and normal brain tissues were fixed with 10% formalin and embedded with paraffin. The clinical samples were collected from clinical operation (Table 3). IHC staining followed standard protocol to evaluate the expression level of IDH1 and BCL3 in human tissues. Staining was graded on a scale of 0-3 according to the intensity and the percentage of immune-positive cells as follows: 0: no staining or <10% positive cells; 1: weak staining in >10% of cells or moderate staining in 10-70% of cells; 2: moderate staining in >70% of cells or strong staining in 10-70% of cells; 3: strong staining in >70% of cells.

**Table 3:** List of databases.

**Patients ID**	**GBM**	**IDH1 mutation**	**Sex**	**Age**	**Antibody**
3732	No	No	Female	64	IDH1
2529	Yes	No	Female	37	IDH1
3022	Yes	No	Female	62	IDH1
2523	No	No	Female	45	BCL3
2027213	Yes	Yes	Male	51	BCL3
2007607	Yes	Yes	Male	48	BCL3
21-911	Yes	No	Male	29	BCL3
21-2133	Yes	No	Male	40	BCL3

### Subcutaneous xenograft studies

All experimental mice were purchased from the Chongqing Medical University. To further study the role of IDH1 in GBM growth, 1 × 10^7^ U87 cells with different treatments were injected into the axilla of 6 weeks old BALB/c nude mice. Tumor growth was determined by length (L) and width (W), which were measured at 4 weeks after the injection. The tumor volume (V), was calculated by V (mm^3^) = (L×W^2^) × 0.5. The use of all mouse samples and the experimental procedures in this study were reviewed and approved by the Ethics Committee of Chongqing University Three Gorges Hospital.

### Statistical analyses

Available samples from TCGA data were adequate because sufficient power using equivalent tests was observed in a previous study [34]. To test for differential expression across two groups (tumor and normal), the R package DESeq2 was used on raw count data [35]. The p-values were adjusted for multiple testing based on the false discovery rate (FDR) according to the Benjamini-Hochberg approach [36]. For comparison of two patient groups, the two-sided Student’s t-test and Wilcoxon-rank sum test was used. For comparisons among multiple-patient groups, one-way ANOVA and Tukey’s honest significant difference (HSD) post hoc tests were used. Distributions of data are shown either as individual data points, as box-and-whisker plots, or as violin plots. The p-values were adjusted according to the Benjamini-Hochberg method.

## RESULTS

### The expression of IDH1 in GBM

**Figure 1:**
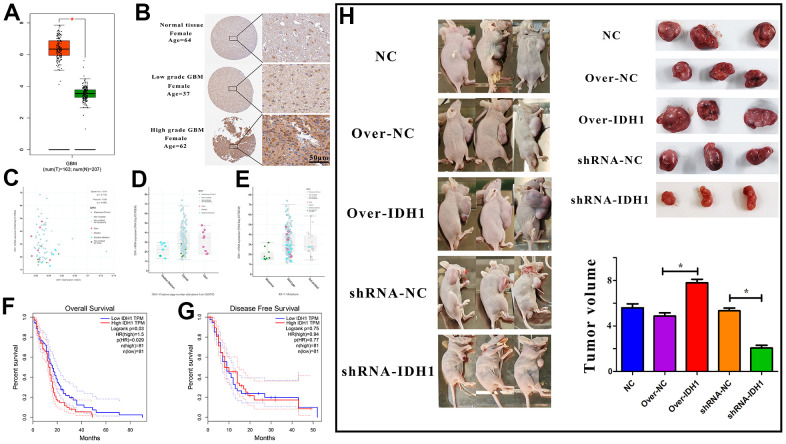
**The expression of IDH1 in GBM.** (**A**) The box figure shows the expression of IDH1 in tumor (n=163) and normal tissues (n=207) with error bars. The red box represents tumor samples and the green one represents normal samples. *, p-value≤0.05. (**B**) The protein level of IDH1 in different GBM grades. The pictures of immunohistochemistry results with different magnifications. (**C**–**E**) The scatter and box diagram shows the correlation between IDH1 mRNA level and methylation, copy number and mutation, respectively. The legend and coefficient of association were listed on the right. (**F**, **G**) The survivorship curves of OV and DFS with a confidence interval, respectively. The red curve represents samples with a higher level of IDH1. Oppositely, the blue one represents samples with a lower level of IDH1. The related parameters were listed on the right. (**H**) Representative images of subcutaneous tumors originated from transfected GBM cells and the corresponding statistical results.

Abundant evidence suggested that IDH1 was functional in GBM progress while being regarded as a significant marker for the tumor classification. According to the RNA-seq of GBM from TCGA, it was detected that the IDH1 was up-regulated in GBM when compared with para-carcinoma tissue (Figure 1A). In addition, the expression level might be positively correlated with malignant degree (Figure 1B). The results may suggest that IDH1 plays a crucial role in tumorigenesis and malignant progression. The gene expression would be influenced by molecular characteristics like methylation, copy number and mutation. However, in GBM, the IDH1 expression was not associated with them. (Figure 1C–1E). Though the mechanism of IDH1 dysregulation was still unclear. The clinical data statistics showed that the lower IDH1 level could improve prognosis (overall survival not disease-free survival) (Figure 1F, 1G). To further confirm the carcinogenesis of IDH1 in GBM, in the subcutaneous xenograft mouse model, once IDH1 expression was increased, the tumor growth rate was accelerated (Figure 1H). Thus, it was speculated that once the IDH1 mutation appeared, the IDH1 enzyme activity would be lost and lead to GBM growth suppression and prognosis improvement.

### The IDH1 mutation in GBM patients

**Figure 2:**
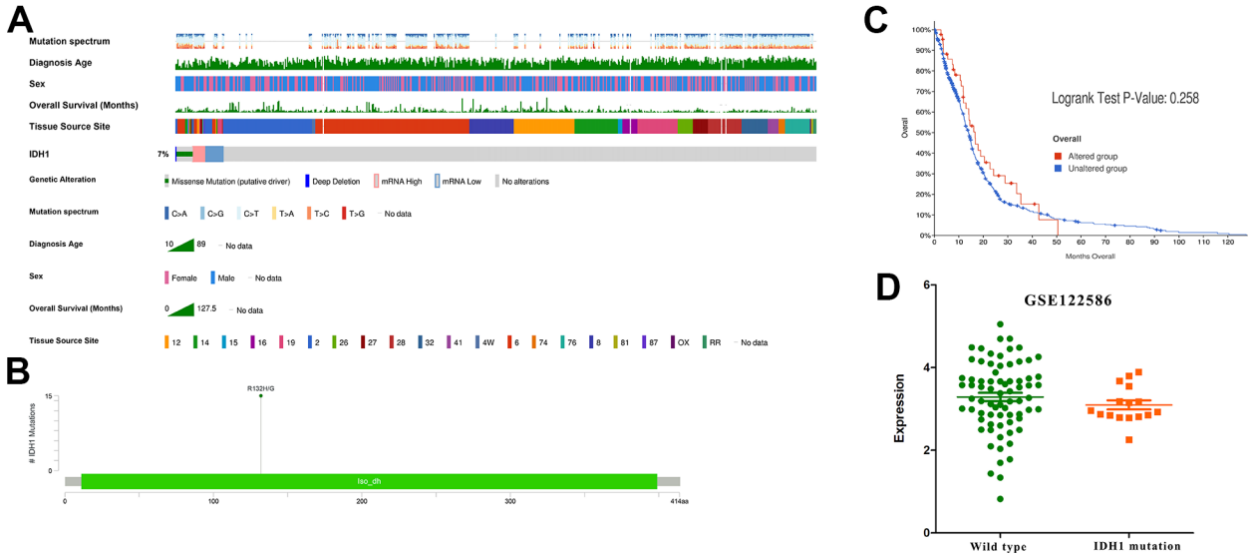
**The IDH1 mutation in GBM.** (**A**) Integrated view of clinical data and IDH1 aberration in GBM patients (370 samples). From top to bottom panels indicate: mutation spectrum, diagnosis age, sex, overall survival (months), tissue source site and mutation symbol of IDH1. The key to the color-coding is at the bottom. (**B**) The schematic diagram of IDH1 mutation site. The abscissa represents the amino acid sequence sites while the ordinate represents the number of mutant samples. (**C**) The survivorship curves of OV. The red curve represents samples with an IDH1 mutation and the blue one represents the wild type. (**D**) A scatter diagram shows the mRNA expression of IDH1 in wild type and mutated samples. The sequencing data (GSE122586) was from the GEO database.

Gene mutation often appears in cancer patients. Through analyzing whole-genome sequencing data of GBM patients from TCGA, the molecular aberrations of IDH1 were detected in nearly 7% of samples (Figure 2A). Among them, about half of patients appear the classic base mutation (R132H/G) (Figure 2B). Statistical survival time showed that once IDH1 mutated, the prognosis will be better without significant difference (p-value=0.258) (Figure 2C). In the previous section, it was demonstrated the expression and mutation of IDH1 were uncorrelated. To further confirm this conclusion, one transcriptome sequencing data (GSE122586) was detected and there was no significance between wild and mutated types (Figure 2D). All results may suggest that the IDH1mutation may suppress GBM development by losing key enzyme activity, like isocitrate binding.

### An enrichment analysis of DEGs regulated by IDH1mutation

**Figure 3:**
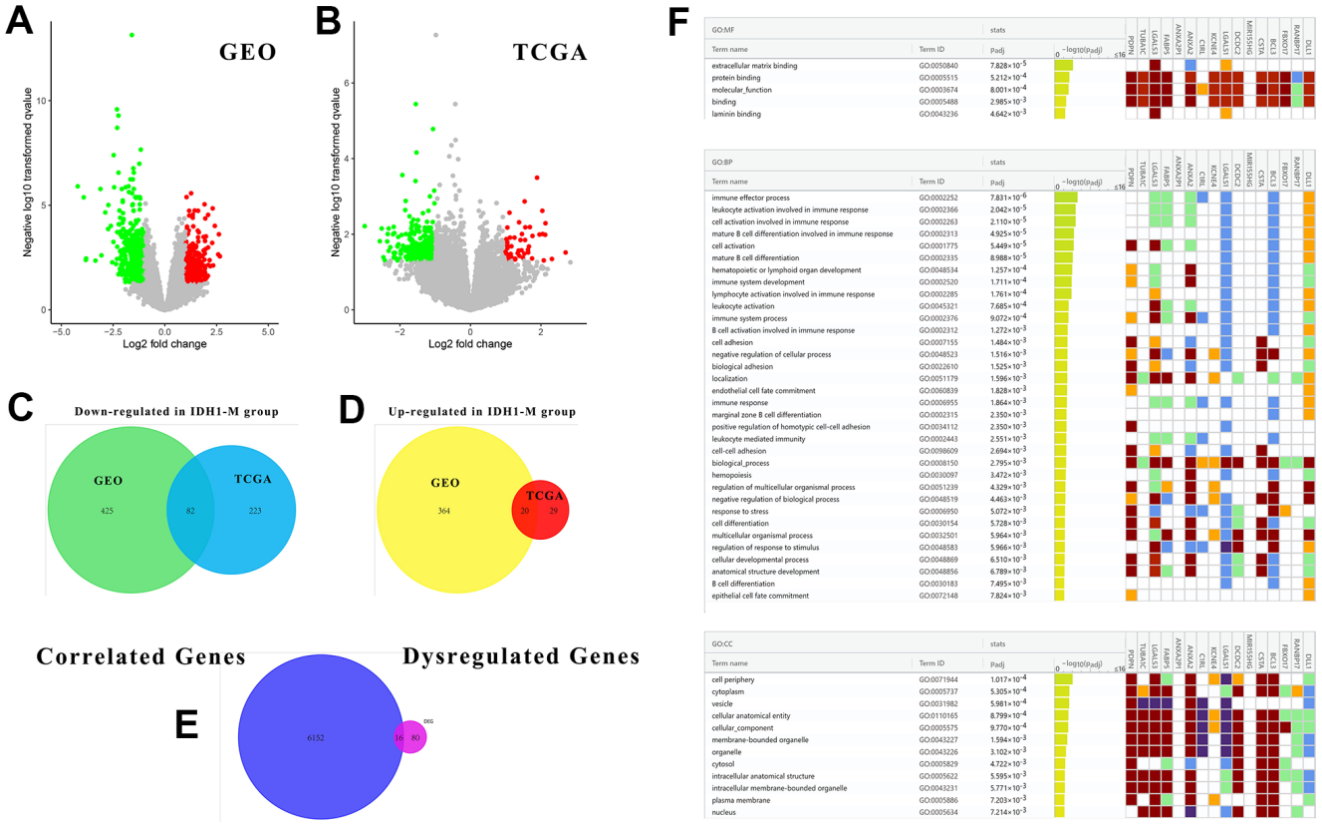
**The associated DEGs with IDH1 and enrichment analysis.** (**A**, **B**) The volcano plots show all DEGs from GEO and TCGA databases, respectively. The red points represent up-regulated genes while the green ones represent down-regulated genes. The gray points represent genes with no significant difference. (**C**, **D**) The Venn diagrams show the up-regulated and down-regulated genes both in GEO and TCGA databases. (**E**) A Venn diagram shows the genes both associated with IDH1 and deregulated by IDH1 mutation. (**F**) A lattice diagram shows the result of GO enrichment analysis of screened genes.

In addition to altering their expression and functions, some mutations may also cause similar changes in downstream or correlated genes. To study the underlying regulatory mechanism of IDH1 mutation, the DEGs in IDH1^mut^ GBM were screened with |log FC|≥1.0 and p-value≤0.05 in both GEO and TCGA datasets (Figure 3A, 3B). To ensure accuracy and increase credibility, the dysregulated gene screened in both databases (n=102) were used for the next analysis (Figure 3C, 3D). Furthermore, there are 6168 genes associated with IDH1 in GBM. Among them, only 16 genes (down-regulated: PDPN, TUBA1C, LGALS3, FABP5, ANXA2P1, ANXA2, C1RL, KCNE4, LGALS1, DCDC2, MIR155HG, CSTA, BCL3 and FBXO17; up-regulated: RANBP17 and DLL1) were dysregulated in IDH1^mut^ GBM simultaneously (Figure 3E and Supplementary Figure 1). Following enrichment analysis for GO function (BP, MF and CC), some immune functions like the immune effector process (adjust p-value=7.831×10^-6^) and immune system development (adjust p-value=1.711×10^-4^) were enriched. It was hypothesized that IDH1 mutations could mediate tumor development via regulating the immune microenvironment.

### The immune correlation of IDH1 mutation in GBM

**Figure 4:**
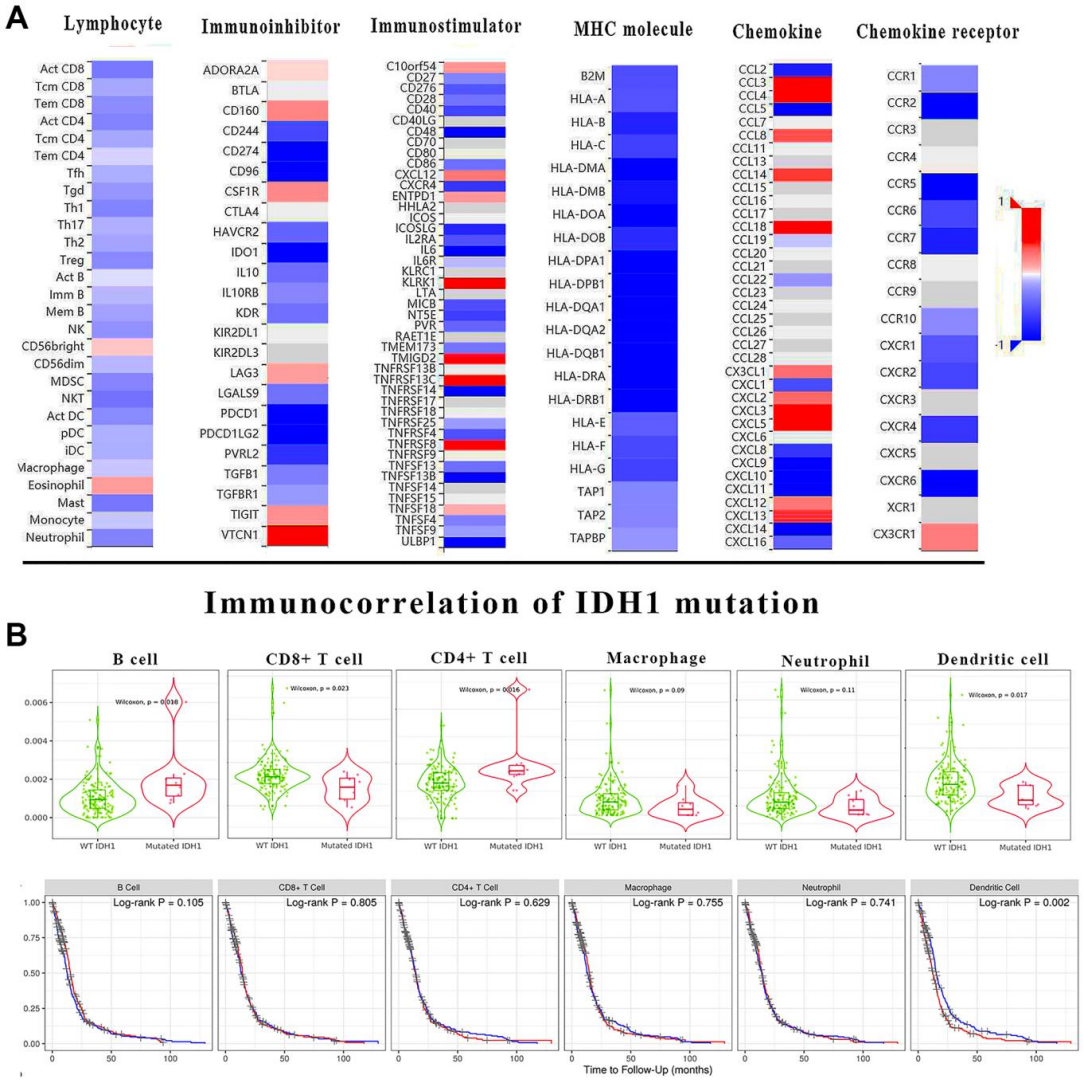
**The immune correlation of IDH1 mutation in GBM.** (**A**) The heat map shows the correlation of IDH1 and various immune indexes. The red box represents positive correlation and the blue one represents negative correlation. (**B**) The violin figures above show the infiltration of main lymphocyte types in wild type or IDH1 mutated samples. The following survivorship curves of OV show the correlation between lymphocyte infiltration and prognosis. The red curves represent the high level of infiltration and the blue ones represent the lower level of infiltration.

In most cancer studies, the immune microenvironment was often considered as an extracellular factor affecting tumor development. Tumor cells can achieve immune escape or suppression by dysregulation of specific genes. It has been demonstrated that IDH1 mutation could be involved in immune functions by regulating the expression of multiple genes. To study the impact of IDH1 mutation on the GBM immune microenvironment more comprehensively, the correlation between IDH1 mutation and lymphocyte infiltration, immune inhibitor, immunostimulator, MHC molecule, chemokine and chemokine receptor was calculated (Figure 4A). Almost lymphocyte infiltrations (Th1 cell) and all MHC molecule (HLA family) expression were negatively correlated with IDH1 mutation. Meanwhile, the expression of some immunostimulators (eg. KLRK1) and chemokine (eg. CCL3) were positively correlated with IDH1 mutation. Lymphocyte infiltration is the most critical event directly regulating tumor immune escape or suppression in the microenvironment. In IDH1 mutated samples, the infiltration of B cell (p-value=0.018) and CD4+ T cell (p-value=0.016) were promoted while CD8+ T cell (p-value=0.023) and dendritic cell (p-value=0.017) were suppressed (Figure 4B). In addition, it was verified that only the infiltration of dendritic cells could significantly alter prognosis (Log-rank P=0.002).

### The clinical character of DEGs correlated with IDH1

**Figure 5:**
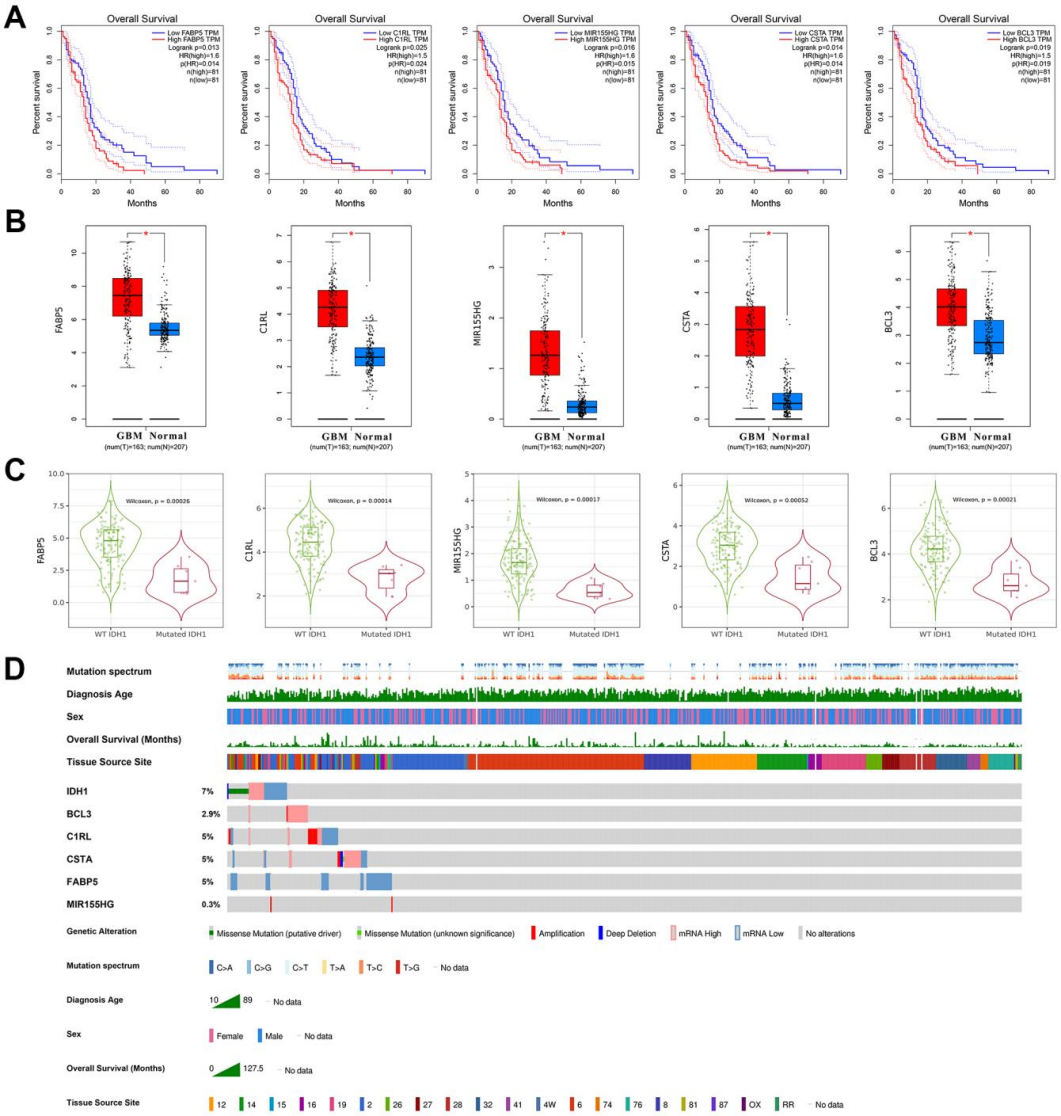
**The clinical character of crucial genes.** (**A**) The survivorship curves of OV with confidence interval. The red curve represents samples with a higher level of the crucial gene. Oppositely, the blue one represents samples with a lower level. The related parameters were listed on the right. (**B**) The box figure shows the expression of genes in tumors (n=163) and normal tissues (n=207) with error bars. The red boxes represent tumor samples and the blue ones represent normal samples. *, p-value≤0.05. (**C**) The violin figures represent the expression level of crucial genes in both wild type and IDH1 mutated samples. (**D**) Integrated view of clinical data and gene aberration in GBM patients (370 samples). From top to bottom panels indicate: mutation spectrum, diagnosis age, sex, overall survival (months), tissue source site and mutation symbol of genes. The key to the color-coding is at the bottom.

As mentioned above, a total of 16 DEGs were identified in IDH1mutated samples which also correlated with IDH1 expression. Among them, only 5 positively related genes (FABP5, C1RL, MIR155HG, CSTA and BCL3) would significantly induce the poor prognosis in GBM patients (p-value≤0.05) (Figure 5A). Notably, in the enrichment analysis above, FABP5, C1RL, CSTA and BCL3 could also participate in various immunology processes, like mature B cell differentiation. Therefore, it was concluded that FABP5, C1RL, MIR155HG, CSTA and BCL3 might mediate the functions of IDH1 mutation in the GBM immune microenvironment. The correlations between the gene expression and lymphocyte infiltrations in the GBM patients were calculated (Supplementary Figure 2). Among them, MIR155HG, a non-coding gene, was not correlated with all types of lymphocyte infiltrations (p-value≥0.05). However, the dendritic cell infiltration was most significantly positively correlated with other genes level (p-value≤0.05). Compared with normal tissues, FABP5, C1RL, MIR155HG, CSTA and BCL3 were significantly increased in GBM (Figure 5B). Nevertheless, in IDH1^mut^ GBM samples, all 5 genes were down-regulated significantly (Figure 5C). Besides MIR155HG, other genes also own higher molecular aberrations frequency in GBM (Figure 5D). It may suggest that in GBM, FABP5, C1RL, CSTA and BCL3 could be regarded as oncogenes like IDH1.

### BCL3 may regulate IDH1 as a transcription factor

**Figure 6:**
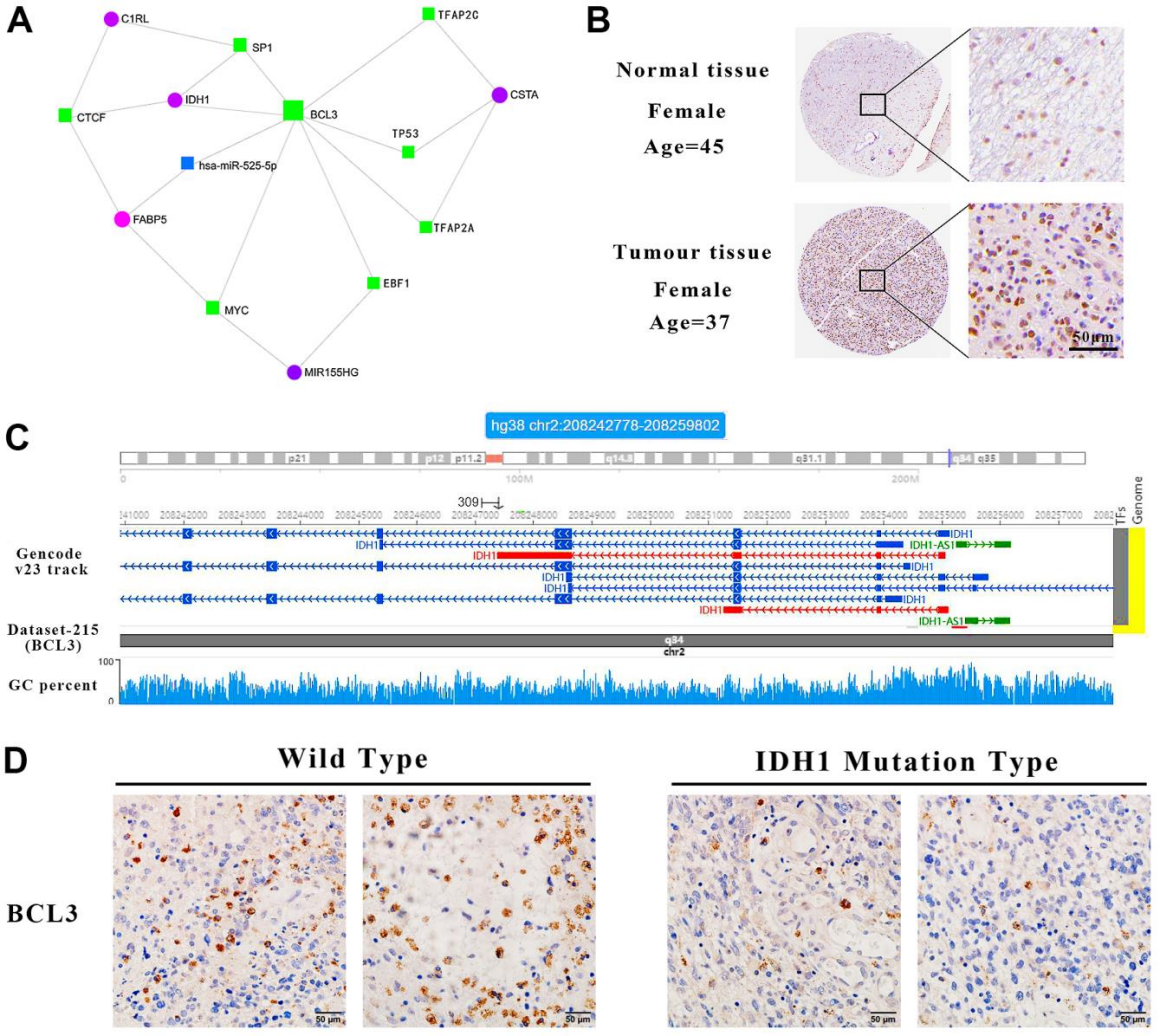
**IDH1 may be a target gene of BCL3.** (**A**) The interact network contains IDH1 and associated genes. The squares represent the transcription factors and the circles represent genes. (**B**) The protein level of BCL3 in normal and GBM tissues. The pictures of immunohistochemistry results with different magnifications. (**C**) Schematic diagram of transcription factor targeting sites. (**D**) The immunohistochemical images of BCL3 in wild type and IDH1 mutation type samples, respectively.

The genes screened previously, including IDH1, perform functions in an interactive network rather than independent manners. Based on the STRING database, an interactive network was constructed for systematically understanding gene functions (Figure 6A). In this network, BCL3 may interact with most genes, including SP1, TP53 and MYC which were defined as crucial oncogenes. Moreover, IDH1 was predicted as a target of BCL3. In GBM tissue, BCL3 was increased and positively correlated with IDH1 (Figure 6B and Supplementary Figure 2). To further confirm that BCL3 could target IDH1, possible action sites were screened by ChIP-sequencing datasets. A total of 4 predicted sites were verified on IDH1 gene, among which one was in the promoter region near the TSS (transcription start site) (Chr2, 208265841-208266367, signal value=4.69), others were in the gene body. The maximum signal peak was identified at Chr2, 208255168-208255438 (signal value=5.50) where has a higher GC percent (Figure 6C). However, whether BCL3 regulates IDH1 and the specific binding site needs to be further studied.

**Figure 7:**
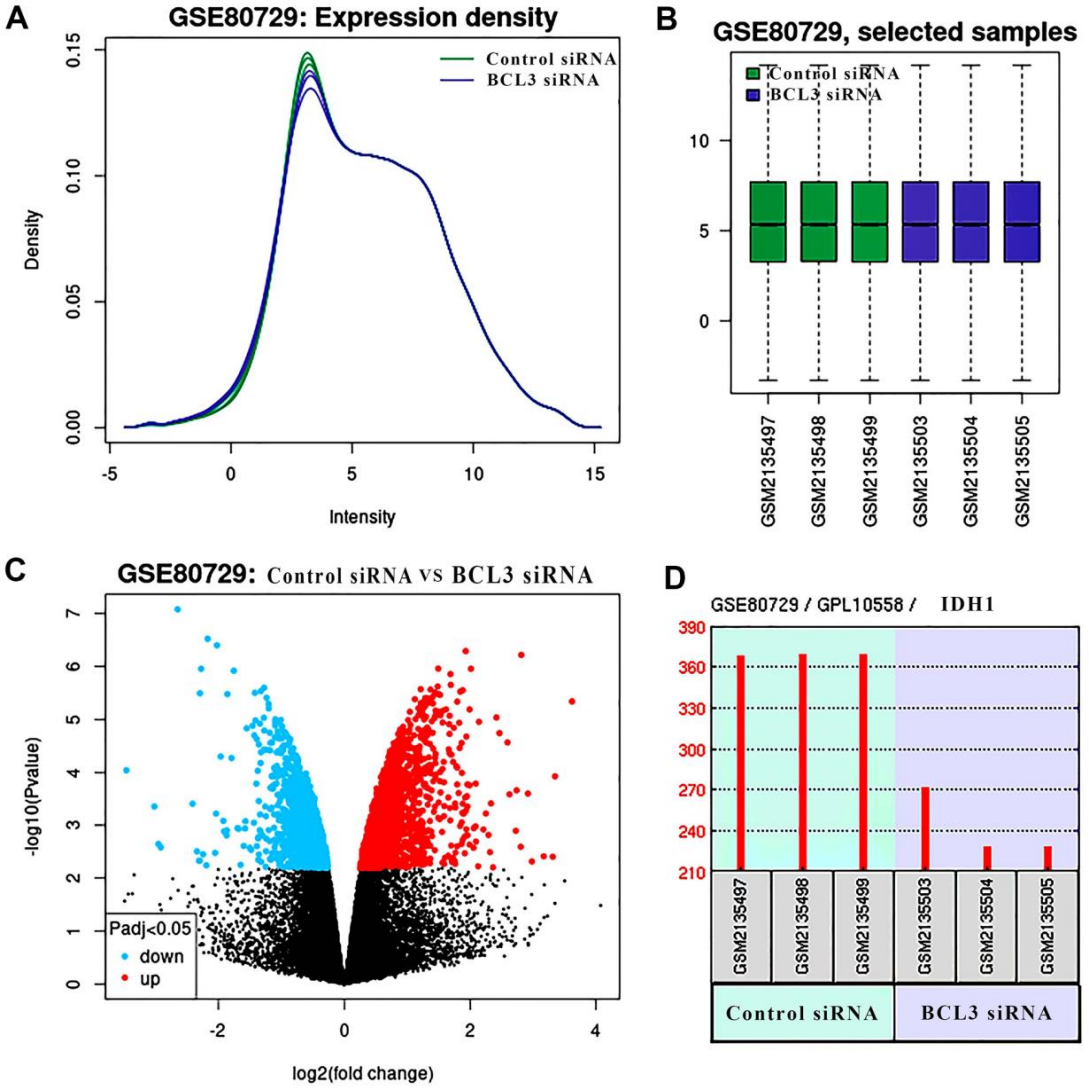
**The DEGs in glioma treat with BCL3 siRNA.** (**A**) The expression density of each sample. (**B**) The box plot shows the distribution of raw read counts. (**C**) The volcano plots show all DEGs of GSE80729 databases. The red points represent up-regulated genes while the blue ones represent down-regulated genes. The black points represent genes with no significant difference. (**D**) The expression of IDH1 in control and BCL3-siRNA group, each group has 3 replicate samples.

To confirm that BCL3 can positively regulate IDH1 in glioma, transcriptome sequencing data containing the glioma cells transfected with siRNA targeting BCL3 or control siRNA were analyzed (Figure 7). After DEG screening, a total of 355 up-regulated genes and 140 down-regulated genes. In addition, the expression of IDH1was downregulated when BCL3 expression was disrupted. The result may further be confirmed that the BCL3 is a transcription factor of IDH1.

### BCL3 and IDH1 in GBM immune microenvironment

**Figure 8:**
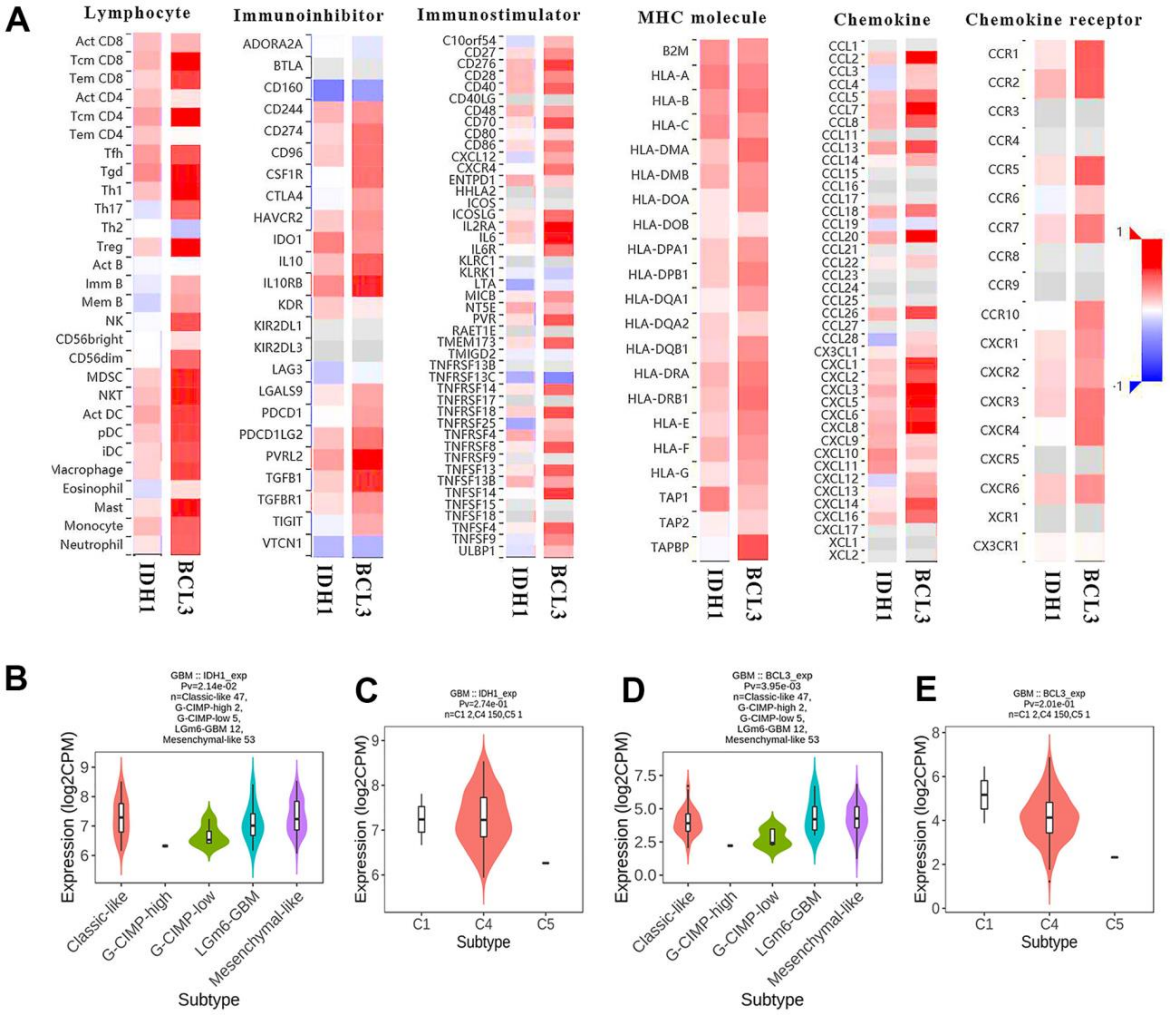
**The immunoscore of BCL3 and IDH1 level.** (**A**) The heat map shows the correlation between IDH1 or BCL3 levels and various immune indexes. The red box represents positive correlation and the blue one represents negative correlation. (**B**–**E**) The violin plots represent the expression of IDH1 and BCL3 in various subtypes.

IDH1 mutation would alter the expression level of BCL3 (Figures 5C, 6D). Meanwhile, BCL3 was predicted to regulate IDH1 as a transcription factor. In addition, both BCL3 and IDH1 might participate in immune functions, like dendritic cell infiltration. To comprehensively study the mechanism of IDH1 and BCL3 in tumor immunity regulation, the correlation between the expression level and various immune events was calculated (Figure 8A). Interestingly, the result showed that BCL3 and IDH1 own a similar correlation of lymphocyte infiltration and immune factor expression. The correlated degrees of BCL3 were higher than those of IDH1 maybe also indicate that in the immune microenvironment, BCL3 owns a larger regulatory capacity. In different subtypes, the similar expression tendency of BCL3 and IDH1 further confirms they have a close relationship.

## DISCUSSION

In a population-based study, the median overall survival of clinically diagnosed GBM patients with IDH1^mut^ was 234 days, significantly longer than IDH1^wt^ patients (141 days; p-value=0.003) [37, 38]. Furthermore, an analysis of GBM patients treated with surgery or/and radiotherapy showed that the mean overall survival of IDH1^mut^ patients was 813 days, longer than patients with IDH1^wt^ GBM (339 days; p-value <0.0001) [39]. However, the underlying mechanism of IDH1 mutation regulating the prognosis of GBM patients was still indistinct. In the present study, via bioinformatics analysis of multiple level sequencing datasets from GEO and TCGA databases, it was demonstrated that IDH1was up-regulated in GBM when compared with adjacent tissue. And the IDH1 mutation did not significantly alter its expression level. However, in Bleeker’s study [15], it was found that NADPH production is hampered in GBM with IDH1 (R132) mutation. Moreover, mutated IDH1 consumes rather than produces NADPH. Thus, likely reducing NADPH levels even further. The low NADPH levels may sensitize GBM to irradiation and chemotherapy, thus explaining the prolonged survival of patients with mutated glioblastoma [40, 41].

Through analysis of transcriptome sequencing data, FABP5, C1RL, MIR155HG, CSTA and BCL3 were screened which were positively correlated with IDH1 and down-regulated in IDH1^mut^ GBMs. However, whether mutated IDH1 regulate these genes expression and the mechanism is still unknown. According to the references [15], once IDH1 mutated, the function would be changed. The tumor metabolite 2-HG could be produced from α-KG by accelerating NADPH consumption [13, 14]. The increased 2-HG competitively suppresses the α-KG-dependent dioxygenases which mediate DNA demethylation [16]. Thus, in GBM, it was speculated that IDH mutation regulates gene expression in an epigenetic manner like DNA hypermethylation rather than direct regulation.

A molecular network including IDH1 and correlated genes was constructed. Among them, lower levels of FABP5, C1RL, MIR155HG, CSTA and BCL3 could improve the prognosis in GBM patients. To understand the function of this network, the enrichment analysis showed that the critical genes (FABP5, C1RL, CSTA and BCL3) could also participate in various immunology processes. It has been studied that in GBM, FABP5 expression correlated with an undifferentiated tumor phenotype as a known tumor-associated antigen that could respond to B cell [42, 43]. It was also confirmed that C1RL was upregulated in GBM, especially mesenchymal GBM and primary GBM. Increased C1RL expression accompanied the IDH1-wt phenotype in both lower-grade glioma (LGG) and GBM. C1RL- associated genes were mainly enriched in biological processes related to the immune response. C1RL expression was also correlated with reduced tumor purity and increased M2 macrophage infiltration [44]. Followed by the immune score, it was hypothesized that the IDH1 may combine with BCL3 to change the GBM immune microenvironment like dendritic cell infiltration. In addition, CSTA upregulation has previously been described in human malignant gliomas: CSTA positive cells in GBM tumor samples were located close to tumor blood vessels, particularly in leukocytes and inflammatory host cells, possibly reflecting the level of inflammatory cells in the tumor tissue. CSTA expression displayed a significant correlation with markers (CD68 and CXCR4) of invasive GBMs [45].

BCL3 was previously found to be a putative proto-oncogene in human cancers and attenuates the efficacy of temozolomide in glioblastoma cells [46]. Only one published study has proved that in the mouse xenograft model, BCL3 inhibited tumor growth via positively regulating STAT3/p-STAT3 and the downstream targets including cyclin D1 [47]. In our study, according to ChIP-sequencing data, multiple BCL3 predicted sites on the IDH1 gene sequence were identified. Therefore, it was speculated that BCL3 might regulate the expression of IDH1 which also needs further research. Through functional enrichment analysis, both IDH1 and BCL3 were found to be involved in multiple immune activities. Previous studies have found that BCL3 could be involved in immune response in various cancer types [48–50]. After calculating immune correlation, lots of similarities were found between both 2 genes. For example, both IDH1 (partial. cor=0.289) and BCL3 (partial. cor=0.512) were positively correlated with dendritic cell infiltration. Dendritic cells are the most powerful antigen-presenting cells. DC-based vaccination has the potential to target and eliminate GBM cells and enhance the responses of malignant cells to the existing therapies with minimal damage to healthy tissues [51]. The efficacy of this therapy can be strengthened in several ways like regulation of regulators in the GBM microenvironment [52]. This will provide a basis for better research on the role of IDH1 mutation in GBM, and has guiding significance for the development of new therapeutic protocols based on the immune function of IDH1 and BCL3.

### Availability of data and material

All data and material were available. The datasets generated and/or analyzed during the current study are available from the corresponding author on reasonable request.

### Ethics approval and consent to participate

The ethical approval about all animal experiment and clinical samples used in our study was obtained from the Chongqing University Three Gorges Hospital.

## Supplementary Material

Supplementary Figures

## References

[1] July J, Patricia D, Gunawan PY, Setiajaya H, Ginting TE, Putra TP, Wuisan Z, Budhiarko D, Masykura N, Prayogi G, Utomo AR, Tandean S, Loe ML. Clinicopathological associations and prognostic values of IDH1 gene mutation, MGMT gene promoter methylation, and PD-L1 expressions in high-grade glioma treated with standard treatment. Pan Afr Med J. 2020; 36:309. 10.11604/pamj.2020.36.309.24831 33282092 PMC7687483

[2] Burgenske DM, Yang J, Decker PA, Kollmeyer TM, Kosel ML, Mladek AC, Caron AA, Vaubel RA, Gupta SK, Kitange GJ, Sicotte H, Youland RS, Remonde D, et al. Molecular profiling of long-term IDH-wildtype glioblastoma survivors. Neuro Oncol. 2019; 21:1458–69. 10.1093/neuonc/noz129 31346613 PMC6827834

[3] Li LC, Zhang M, Feng YK, Wang XJ. IDH1-R132H Suppresses Glioblastoma Malignancy through FAT1-ROS-HIF-1α Signaling. Neurol India. 2020; 68:1050–8. 33109851 10.4103/0028-3886.294557

[4] Parsons DW, Jones S, Zhang X, Lin JC, Leary RJ, Angenendt P, Mankoo P, Carter H, Siu IM, Gallia GL, Olivi A, McLendon R, Rasheed BA, et al. An integrated genomic analysis of human glioblastoma multiforme. Science. 2008; 321:1807–12. 10.1126/science.1164382 18772396 PMC2820389

[5] Karsy M, Guan J, Cohen AL, Jensen RL, Colman H. New Molecular Considerations for Glioma: IDH, ATRX, BRAF, TERT, H3 K27M. Curr Neurol Neurosci Rep. 2017; 17:19. 10.1007/s11910-017-0722-5 28271343

[6] Su X, Sun H, Chen N, Roberts N, Yang X, Wang W, Li J, Huang X, Gong Q, Yue Q. A radiomics-clinical nomogram for preoperative prediction of IDH1 mutation in primary glioblastoma multiforme. Clin Radiol. 2020; 75:963.e7–15. 10.1016/j.crad.2020.07.036 32921406

[7] Polívka J Jr, Pešta M, Pitule P, Hes O, Holubec L, Polívka J, Kubíková T, Tonar Z. IDH1 mutation is associated with lower expression of VEGF but not microvessel formation in glioblastoma multiforme. Oncotarget. 2018; 9:16462–76. 10.18632/oncotarget.24536 29662659 PMC5893254

[8] Philip B, Yu DX, Silvis MR, Shin CH, Robinson JP, Robinson GL, Welker AE, Angel SN, Tripp SR, Sonnen JA, VanBrocklin MW, Gibbons RJ, Looper RE, et al. Mutant IDH1 Promotes Glioma Formation *In Vivo*. Cell Rep. 2018; 23:1553–64. 10.1016/j.celrep.2018.03.133 29719265 PMC6032974

[9] Molenaar RJ, Wilmink JW. IDH1/2 Mutations in Cancer Stem Cells and Their Implications for Differentiation Therapy. J Histochem Cytochem. 2022; 70:83–97. 10.1369/00221554211062499 34967233 PMC8721574

[10] Thirumal Kumar D, Sneha P, Uppin J, Usha S, George Priya Doss C. Investigating the Influence of Hotspot Mutations in Protein-Protein Interaction of IDH1 Homodimer Protein: A Computational Approach. Adv Protein Chem Struct Biol. 2018; 111:243–61. 10.1016/bs.apcsb.2017.08.002 29459034

[11] Juritz EI, Bascur JP, Almonacid DE, González-Nilo FD. Novel Insights for Inhibiting Mutant Heterodimer IDH1wt-R132H in Cancer: An *In-Silico* Approach. Mol Diagn Ther. 2018; 22:369–80. 10.1007/s40291-018-0331-2 29651790

[12] Zhao S, Lin Y, Xu W, Jiang W, Zha Z, Wang P, Yu W, Li Z, Gong L, Peng Y, Ding J, Lei Q, Guan KL, Xiong Y. Glioma-derived mutations in IDH1 dominantly inhibit IDH1 catalytic activity and induce HIF-1alpha. Science. 2009; 324:261–5. 10.1126/science.1170944 19359588 PMC3251015

[13] Dang L, White DW, Gross S, Bennett BD, Bittinger MA, Driggers EM, Fantin VR, Jang HG, Jin S, Keenan MC, Marks KM, Prins RM, Ward PS, et al. Cancer-associated IDH1 mutations produce 2-hydroxyglutarate. Nature. 2009; 462:739–44. 10.1038/nature08617 19935646 PMC2818760

[14] Ward PS, Patel J, Wise DR, Abdel-Wahab O, Bennett BD, Coller HA, Cross JR, Fantin VR, Hedvat CV, Perl AE, Rabinowitz JD, Carroll M, Su SM, et al. The common feature of leukemia-associated IDH1 and IDH2 mutations is a neomorphic enzyme activity converting alpha-ketoglutarate to 2-hydroxyglutarate. Cancer Cell. 2010; 17:225–34. 10.1016/j.ccr.2010.01.020 20171147 PMC2849316

[15] Bleeker FE, Atai NA, Lamba S, Jonker A, Rijkeboer D, Bosch KS, Tigchelaar W, Troost D, Vandertop WP, Bardelli A, Van Noorden CJ. The prognostic IDH1(R132) mutation is associated with reduced NADP+-dependent IDH activity in glioblastoma. Acta Neuropathol. 2010; 119:487–94. 10.1007/s00401-010-0645-6 20127344 PMC2841753

[16] Xu W, Yang H, Liu Y, Yang Y, Wang P, Kim SH, Ito S, Yang C, Wang P, Xiao MT, Liu LX, Jiang WQ, Liu J, et al. Oncometabolite 2-hydroxyglutarate is a competitive inhibitor of α-ketoglutarate-dependent dioxygenases. Cancer Cell. 2011; 19:17–30. 10.1016/j.ccr.2010.12.014 21251613 PMC3229304

[17] Noushmehr H, Weisenberger DJ, Diefes K, Phillips HS, Pujara K, Berman BP, Pan F, Pelloski CE, Sulman EP, Bhat KP, Verhaak RG, Hoadley KA, Hayes DN, et al, and Cancer Genome Atlas Research Network. Identification of a CpG island methylator phenotype that defines a distinct subgroup of glioma. Cancer Cell. 2010; 17:510–22. 10.1016/j.ccr.2010.03.017 20399149 PMC2872684

[18] Viswanath P, Radoul M, Izquierdo-Garcia JL, Ong WQ, Luchman HA, Cairncross JG, Huang B, Pieper RO, Phillips JJ, Ronen SM. 2-Hydroxyglutarate-Mediated Autophagy of the Endoplasmic Reticulum Leads to an Unusual Downregulation of Phospholipid Biosynthesis in Mutant IDH1 Gliomas. Cancer Res. 2018; 78:2290–304. 10.1158/0008-5472.CAN-17-2926 29358170 PMC5932252

[19] Sasaki M, Knobbe CB, Itsumi M, Elia AJ, Harris IS, Chio II, Cairns RA, McCracken S, Wakeham A, Haight J, Ten AY, Snow B, Ueda T, et al. D-2-hydroxyglutarate produced by mutant IDH1 perturbs collagen maturation and basement membrane function. Genes Dev. 2012; 26:2038–49. 10.1101/gad.198200.112 22925884 PMC3444730

[20] Luchman HA, Stechishin OD, Dang NH, Blough MD, Chesnelong C, Kelly JJ, Nguyen SA, Chan JA, Weljie AM, Cairncross JG, Weiss S. An *in vivo* patient-derived model of endogenous IDH1-mutant glioma. Neuro Oncol. 2012; 14:184–91. 10.1093/neuonc/nor207 22166263 PMC3266388

[21] Choi HJ, Lee JM, Kim H, Nam HJ, Shin HJ, Kim D, Ko E, Noh DY, Kim KI, Kim JH, Baek SH. Bcl3-dependent stabilization of CtBP1 is crucial for the inhibition of apoptosis and tumor progression in breast cancer. Biochem Biophys Res Commun. 2010; 400:396–402. 10.1016/j.bbrc.2010.08.084 20800578

[22] Pallares J, Martínez-Guitarte JL, Dolcet X, Llobet D, Rue M, Palacios J, Prat J, Matias-Guiu X. Abnormalities in the NF-kappaB family and related proteins in endometrial carcinoma. J Pathol. 2004; 204:569–77. 10.1002/path.1666 15481028

[23] Tu K, Liu Z, Yao B, Xue Y, Xu M, Dou C, Yin G, Wang J. BCL-3 promotes the tumor growth of hepatocellular carcinoma by regulating cell proliferation and the cell cycle through cyclin D1. Oncol Rep. 2016; 35:2382–90. 10.3892/or.2016.4616 26882953

[24] Puvvada SD, Funkhouser WK, Greene K, Deal A, Chu H, Baldwin AS, Tepper JE, O’Neil BH. NF-kB and Bcl-3 activation are prognostic in metastatic colorectal cancer. Oncology. 2010; 78:181–8. 10.1159/000313697 20414006 PMC2914399

[25] Liu Z, Jiang Y, Hou Y, Hu Y, Cao X, Tao Y, Xu C, Liu S, Wang S, Wang L, Shi Y, Siebenlist U, Zhang X. The IκB family member Bcl-3 stabilizes c-Myc in colorectal cancer. J Mol Cell Biol. 2013; 5:280–2. 10.1093/jmcb/mjt020 23794716

[26] Zhang B, Shen R, Cheng S, Feng L. Immune microenvironments differ in immune characteristics and outcome of glioblastoma multiforme. Cancer Med. 2019; 8:2897–907. 10.1002/cam4.2192 31038851 PMC6558448

[27] Tian Y, Ma L, Cai X, Zhu J. Statistical Method Based on Bayes-Type Empirical Score Test for Assessing Genetic Association with Multilocus Genotype Data. Int J Genomics. 2020; 2020:4708152. 10.1155/2020/4708152 32455126 PMC7229558

[28] Chen X. False discovery rate control for multiple testing based on discrete p-values. Biom J. 2020; 62:1060–79. 10.1002/bimj.201900163 31958180

[29] Chen L, Lu D, Sun K, Xu Y, Hu P, Li X, Xu F. Identification of biomarkers associated with diagnosis and prognosis of colorectal cancer patients based on integrated bioinformatics analysis. Gene. 2019; 692:119–25. 10.1016/j.gene.2019.01.001 30654001

[30] Bindea G, Mlecnik B, Hackl H, Charoentong P, Tosolini M, Kirilovsky A, Fridman WH, Pagès F, Trajanoski Z, Galon J. ClueGO: a Cytoscape plug-in to decipher functionally grouped gene ontology and pathway annotation networks. Bioinformatics. 2009; 25:1091–3. 10.1093/bioinformatics/btp101 19237447 PMC2666812

[31] Huang DW, Sherman BT, Lempicki RA. Systematic and integrative analysis of large gene lists using DAVID bioinformatics resources. Nat Protoc. 2009; 4:44–57. 10.1038/nprot.2008.211 19131956

[32] Li R, Yang YE, Yin YH, Zhang MY, Li H, Qu YQ. Methylation and transcriptome analysis reveal lung adenocarcinoma-specific diagnostic biomarkers. J Transl Med. 2019; 17:324. 10.1186/s12967-019-2068-z 31558162 PMC6764142

[33] Ladas EJ, Blonquist TM, Puligandla M, Orjuela M, Stevenson K, Cole PD, Athale UH, Clavell LA, Leclerc JM, Laverdiere C, Michon B, Schorin MA, Greene Welch J, et al. Protective Effects of Dietary Intake of Antioxidants and Treatment-Related Toxicity in Childhood Leukemia: A Report From the DALLT Cohort. J Clin Oncol. 2020; 38:2151–9. 10.1200/JCO.19.02555 32330103

[34] Angelova M, Charoentong P, Hackl H, Fischer ML, Snajder R, Krogsdam AM, Waldner MJ, Bindea G, Mlecnik B, Galon J, Trajanoski Z. Characterization of the immunophenotypes and antigenomes of colorectal cancers reveals distinct tumor escape mechanisms and novel targets for immunotherapy. Genome Biol. 2015; 16:64. 10.1186/s13059-015-0620-6 25853550 PMC4377852

[35] Wang Z, Tang W, Yuan J, Qiang B, Han W, Peng X. Integrated Analysis of RNA-Binding Proteins in Glioma. Cancers (Basel). 2020; 12:892. 10.3390/cancers12040892 32272554 PMC7226056

[36] Kocak M, Mozhui K. An Application of the Bayesian Periodicity Test to Identify Diurnal Rhythm Genes in the Brain. IEEE/ACM Trans Comput Biol Bioinform. 2020; 17:47–55. 10.1109/TCBB.2018.2859971 30047896

[37] Park YW, Han K, Ahn SS, Bae S, Choi YS, Chang JH, Kim SH, Kang SG, Lee SK. Prediction of IDH1-Mutation and 1p/19q-Codeletion Status Using Preoperative MR Imaging Phenotypes in Lower Grade Gliomas. AJNR Am J Neuroradiol. 2018; 39:37–42. 10.3174/ajnr.A5421 29122763 PMC7410710

[38] de Quintana-Schmidt C, Alvarez-Holzapfel MJ, Nomdedeu-Guinot J, Bague-Rosell S, Gallego-Rubio O, Leidinger A, Salgado-Lopez L, Molet-Teixidó J. [Isocitrate dehydrogenase type I mutation as a prognostic factor in glioblastoma and a literature review]. Neurocirugia (Astur). 2015; 26:276–83. 10.1016/j.neucir.2015.04.001 26194445

[39] Nobusawa S, Watanabe T, Kleihues P, Ohgaki H. IDH1 mutations as molecular signature and predictive factor of secondary glioblastomas. Clin Cancer Res. 2009; 15:6002–7. 10.1158/1078-0432.CCR-09-0715 19755387

[40] Atai NA, Renkema-Mills NA, Bosman J, Schmidt N, Rijkeboer D, Tigchelaar W, Bosch KS, Troost D, Jonker A, Bleeker FE, Miletic H, Bjerkvig R, De Witt Hamer PC, Van Noorden CJ. Differential activity of NADPH-producing dehydrogenases renders rodents unsuitable models to study IDH1R132 mutation effects in human glioblastoma. J Histochem Cytochem. 2011; 59:489–503. 10.1369/0022155411400606 21527585 PMC3201175

[41] Baldewpersad Tewarie NM, Burgers IA, Dawood Y, den Boon HC, den Brok MG, Klunder JH, Koopmans KB, Rademaker E, van den Broek HB, van den Bersselaar SM, Witjes JJ, Van Noorden CJ, Atai NA. NADP+ -dependent IDH1 R132 mutation and its relevance for glioma patient survival. Med Hypotheses. 2013; 80:728–31. 10.1016/j.mehy.2013.02.022 23541771

[42] Mock A, Warta R, Geisenberger C, Bischoff R, Schulte A, Lamszus K, Stadler V, Felgenhauer T, Schichor C, Schwartz C, Matschke J, Jungk C, Ahmadi R, et al. Printed peptide arrays identify prognostic TNC serumantibodies in glioblastoma patients. Oncotarget. 2015; 6:13579–90. 10.18632/oncotarget.3791 25944688 PMC4537035

[43] Campos B, Centner FS, Bermejo JL, Ali R, Dorsch K, Wan F, Felsberg J, Ahmadi R, Grabe N, Reifenberger G, Unterberg A, Burhenne J, Herold-Mende C. Aberrant expression of retinoic acid signaling molecules influences patient survival in astrocytic gliomas. Am J Pathol. 2011; 178:1953–64. 10.1016/j.ajpath.2011.01.051 21514413 PMC3081142

[44] Wang J, Tong L, Lin G, Wang H, Zhang L, Yang X. Immunological and clinicopathological characteristics of C1RL in 2120 glioma patients. BMC Cancer. 2020; 20:931. 10.1186/s12885-020-07436-6 32993564 PMC7526369

[45] Gole B, Huszthy PC, Popović M, Jeruc J, Ardebili YS, Bjerkvig R, Lah TT. The regulation of cysteine cathepsins and cystatins in human gliomas. Int J Cancer. 2012; 131:1779–89. 10.1002/ijc.27453 22287159

[46] Wu L, Bernal GM, Cahill KE, Pytel P, Fitzpatrick CA, Mashek H, Weichselbaum RR, Yamini B. BCL3 expression promotes resistance to alkylating chemotherapy in gliomas. Sci Transl Med. 2018; 10:eaar2238. 10.1126/scitranslmed.aar2238 29973405 PMC6613219

[47] Wu J, Li L, Jiang G, Zhan H, Wang N. B-cell CLL/lymphoma 3 promotes glioma cell proliferation and inhibits apoptosis through the oncogenic STAT3 pathway. Int J Oncol. 2016; 49:2471–9. 10.3892/ijo.2016.3729 27748795

[48] Zou Y, Uddin MM, Padmanabhan S, Zhu Y, Bu P, Vancura A, Vancurova I. The proto-oncogene Bcl3 induces immune checkpoint PD-L1 expression, mediating proliferation of ovarian cancer cells. J Biol Chem. 2018; 293:15483–96. 10.1074/jbc.RA118.004084 30135206 PMC6177577

[49] Kang S, Yun J, Kim DY, Jung SY, Kim YJ, Park JH, Ji ST, Jang WB, Ha J, Kim JH, Baek SH, Kwon SM. Adequate concentration of B cell leukemia/lymphoma 3 (Bcl3) is required for pluripotency and self-renewal of mouse embryonic stem cells via downregulation of Nanog transcription. BMB Rep. 2018; 51:92–7. 10.5483/bmbrep.2018.51.2.219 29335071 PMC5836563

[50] Kim YM, Sharma N, Nyborg JK. The proto-oncogene Bcl3, induced by Tax, represses Tax-mediated transcription via p300 displacement from the human T-cell leukemia virus type 1 promoter. J Virol. 2008; 82:11939–47. 10.1128/JVI.01356-08 18815299 PMC2583681

[51] Marsh JC, Goldfarb J, Shafman TD, Diaz AZ. Current status of immunotherapy and gene therapy for high-grade gliomas. Cancer Control. 2013; 20:43–8. 10.1177/107327481302000107 23302906

[52] Yang L, Guo G, Niu XY, Liu J. Dendritic Cell-Based Immunotherapy Treatment for Glioblastoma Multiforme. Biomed Res Int. 2015; 2015:717530. 10.1155/2015/717530 26167495 PMC4488155

