# Supplementary material for: The immune regulation of BCL3 in glioblastoma with mutated IDH1
Source: Aging (Albany NY). 2022 Apr 29;14(9):3856–73. doi: 10.18632/aging.204048 (PMC9134951; doi:10.18632/aging.204048)
Supplement: Supplementary Figures [file aging-14-204048-s001.pdf]

SUPPLEMENTARY FIGURES

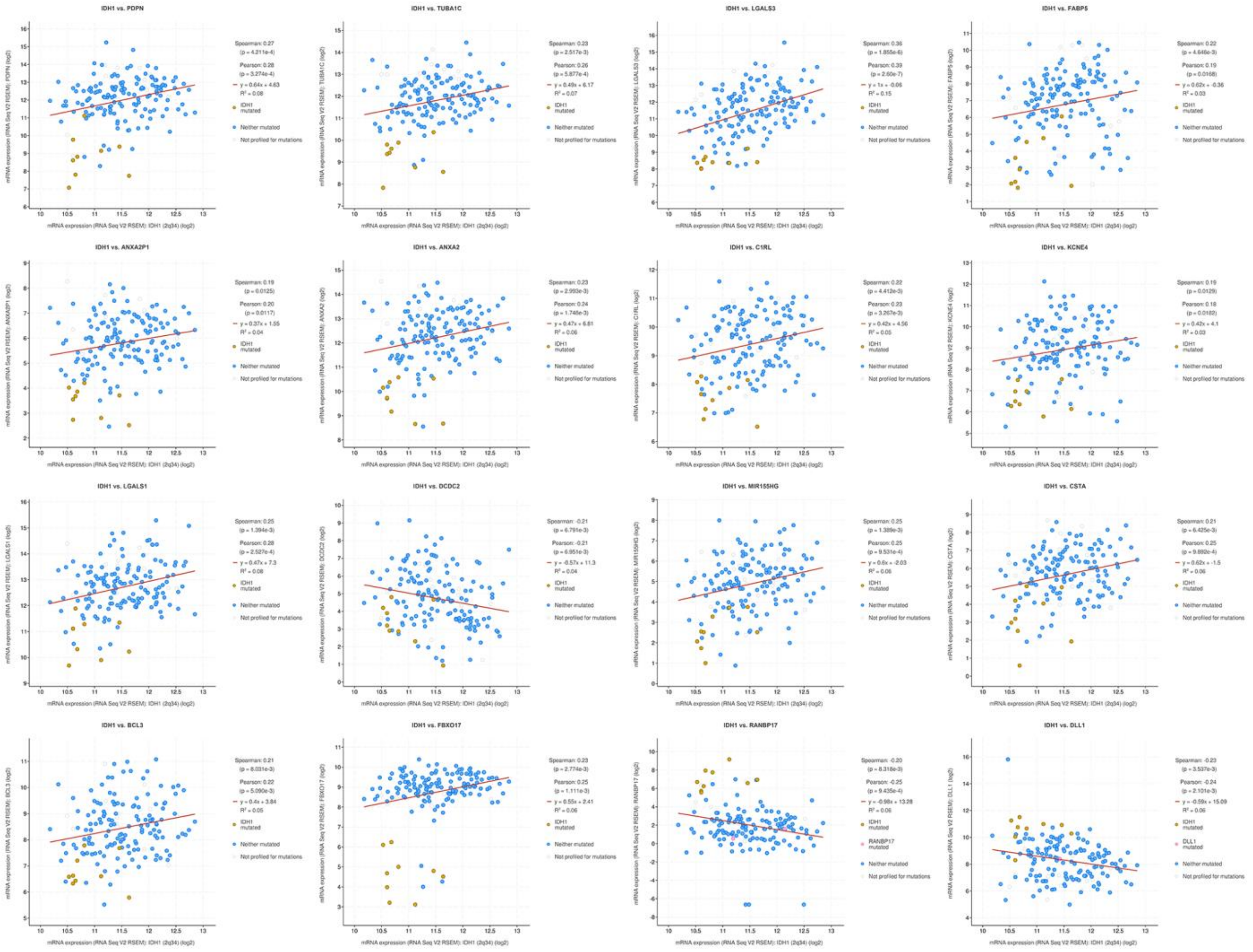

Supplementary Figure 1. The expression level correlation between IDH1 and DEGs. The red line represents the linear regression equation, and the correlation was calculated by Spearman and Pearson algorithms simultaneously (with p-value). The yellow dots represent samples with IDH1 mutations, the blue ones represent samples neither mutated and the white ones represent no profiled for mutations.

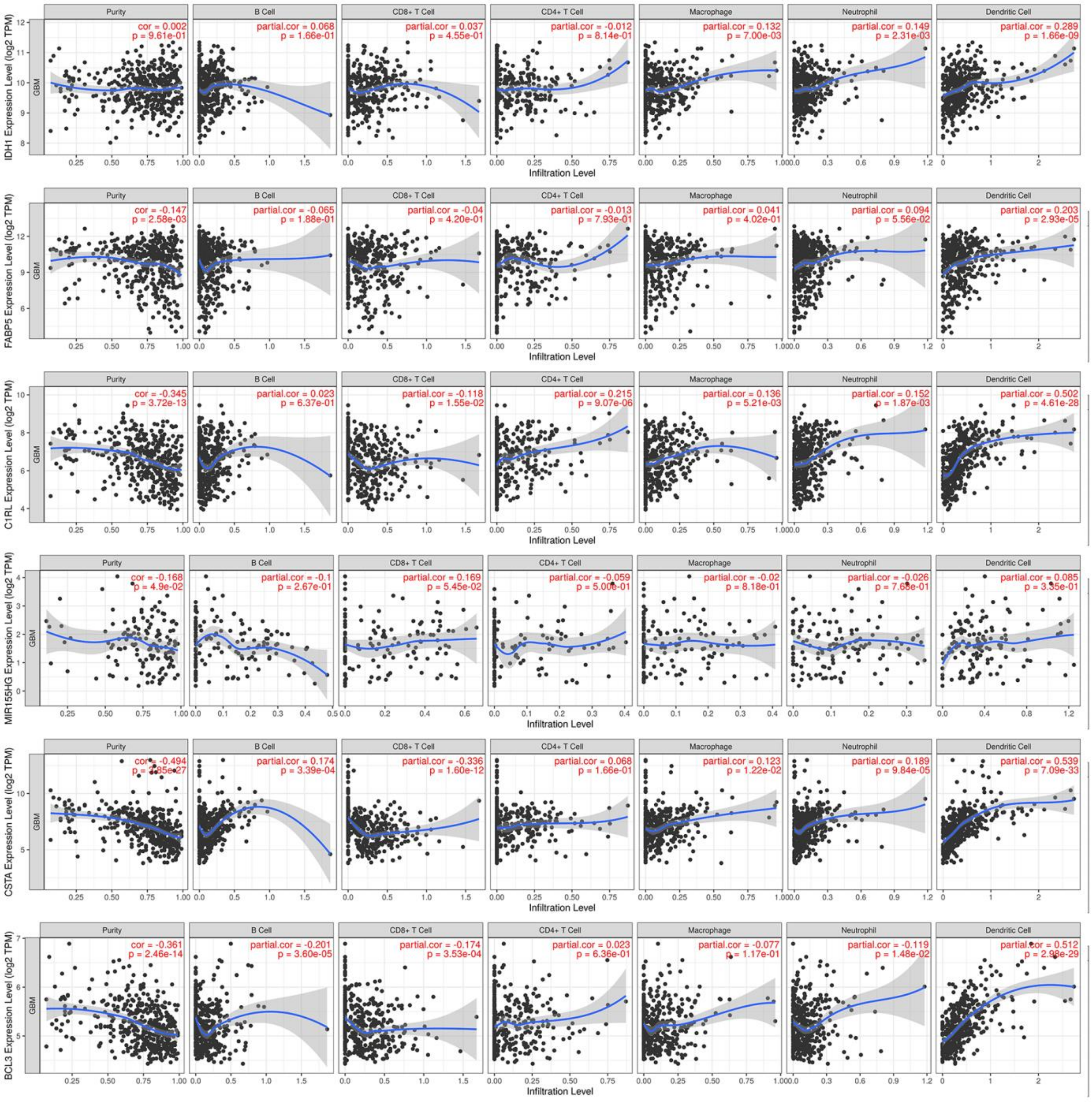

Supplementary Figure 2. The immune correlation of key genes. The correlation between expression levels of key genes and tumor purity or 6 classical immune cell infiltrations was calculated. Each dot represents a tumor sample. The blue line represents the correlation curve.
